# Clec7a expression in inflammatory macrophages orchestrates progression of acute kidney injury

**DOI:** 10.3389/fimmu.2022.1008727

**Published:** 2022-09-15

**Authors:** Yaqiong Wang, Xianzhe Li, Xialian Xu, Jinbo Yu, Xiaohong Chen, Xuesen Cao, Jianzhou Zou, Bo Shen, Xiaoqiang Ding

**Affiliations:** ^1^ Department of Nephrology, Zhongshan Hospital, Fudan University, Shanghai, China; ^2^ Shanghai Medical Center of Kidney, Shanghai, China; ^3^ Department of Nephrology, Shanghai Institute of Kidney and Dialysis, Shanghai, China; ^4^ Shanghai Key Laboratory of Kidney and Blood Purification, Shanghai, China; ^5^ Hemodialysis Quality Control Center of Shanghai, Shanghai, China

**Keywords:** acute kidney injury (AKI), ischemia/reperfusion injury (IRI), macrophages, Clec7a, M1 macrophages, M2 macrophages

## Abstract

Acute kidney injury (AKI) is associated with high risk of mortality, post-disease renal fibrosis, kidney dysfunction and renal failure. Renal macrophages play a key role in the pathogenesis (M1 subpopulation), healing and remodeling (M2 subpopulation) in AKI and, thus, have been a promising target for clinical treatment of AKI. Here, in a mouse renal ischemia/reperfusion injury (IRI) model for AKI, we showed that renal macrophages could be further classified into Clec7a+ M1 macrophages, Clec7a- M1 macrophages, Clec7a+ M2 macrophages and Clec7a- M2 macrophages, representing distinct macrophage populations with different functionality. Interestingly, Clec7a+ M1 macrophages exhibited potent pro-inflammatory and phagocytic effects compared to Clec7a- M1 macrophages, while Clec7a- M2 macrophages exhibited better proliferating and migrating potential, which is critical for their role in tissue repairing after injury. These data from mice were further strengthened by bioinformatics analyses using published database. *In vivo*, combined expression of Clec7a in M1 macrophages and depletion of Clec7a in M2 macrophages significantly improved the renal function after IRI-AKI. Together, our data suggest that Clec7a is crucial for the fine regulation of macrophage phenotype during AKI and could be a novel target for boosting clinical therapy.

## Introduction

Acute kidney injury (AKI) is characterized with a rapid renal dysfunction, post-disease renal fibrosis and renal failure (1). Ischemia/reperfusion injury (IRI) is one of the major causes of AKI (2–5). The high mortality rate of AKI is largely due to the lack of early diagnosis methods and the lack of specific treatments (6). IRI induces innate immune responses, activate complement and innate immune cells, and release a large number of damage-related molecules, cytokines, chemokines and inflammatory mediators, which in turn recruit and activate immune inflammatory cells such as macrophages into the microenvironment of renal injury, causing renal tubular damage, the necrosis and apoptosis of tubular epithelial cells (TECs) (7). Phagocytic classic macrophages (M1 macrophages) and phagocytic TECs that synergistically phagocytose the surrounding Injured cells are the key innate immune cells regulating injury, repair, or fibrotic renal tissue remodeling (8). At the time of inflammation clearance, tissue regeneration and repair involving alternatively activated macrophages (M2 macrophages) are also initiated and progressed (9).

Since renal macrophages play a key role in the pathogenesis (M1 subpopulation), healing and remodeling (M2 subpopulation) in AKI and, thus, have been a promising target for clinical treatment of AKI (10). Lipopolysaccharide (LPS) is the major components of the cell wall of bacteria (11). The LPS-induced macrophages are M1-polarized and have irregular cell morphology (11). On the other hand, macrophages undergo M2-type polarization upon stimulation with interleukin (IL)-4/13 (11). IL-4 is a multifunctional cytokine that reduces the production of pro-inflammatory cytokines and plays a key role in regulating immune and inflammatory responses (11). Both M1 and M2 macrophages as well as their transition are critical for AKI (12). However, the exact molecular phenotypic regulation of renal macrophages is not known.

C-type lectin domain family 7 member A (Clec7a, or Dectin-1) is a transmembrane protein containing an intracellular immunoreceptor tyrosine-based activation (ITAM)-like motif and an extracellular C-type lectin-like domain for recognition (13). Clec7a is expressed by macrophages and some other immune cells and is believed to control the innate immune responses to pathogens and the phagocytotic properties (14–16), through regulating phagocytosis and production of reactive oxygen species (ROS) (17). Moreover, a recent study has shown that Clec7a is critical for macrophage polarization during renal interstitial fibrosis (18). However, the expression pattern of Clec7a in macrophages, as well as its relation to AKI, has not been studied before and thus addressed in the current study.

## Materials and methods

### Ethical approval of research protocols

This study including the animal protocols has been approved by Fudan University Research Ethic Committee and Fudan University Animal Care and Use Committee.

### Animal experiments

C57/Bl6 mice (SLAC Laboratory Animal, Shanghai, China) were kept on a 12-hour light/12-hour dark cycle in a strictly temperature-controlled room, allowed for ad libitum access to water and food. The male and female mice were randomly and equally divided into experimental groups of 6 each (3 males and 3 females): 1) Group 1: Sham, 2) Group 2: IRI, 3) Group 3: IRI and *in situ* administration of adeno-associated virus (AAV) serotype 2 carrying pCD86-Scramble (Scr) and AAV serotype 2 carrying pCD163-Scr, 4) Group 4: IRI and *in situ* administration of AAV serotype 2 carrying pCD86-Clec7a and AAV serotype 2 carrying pCD163-Scr, 5) Group 5: IRI and *in situ* administration of AAV serotype 2 carrying pCD86-Scr and AAV serotype 2 carrying pCD163-siRNA for Clec7a (si-Clec7a), 6) Group 6: IRI and *in situ* administration of AAV serotype 2 carrying pCD86-Clec7a and AAV serotype 2 carrying pCD163-si-Clec7a. For IRI model, mice received a 12 hours’ fasting and then anesthetized with 2.5% chloral hydrate. A 12-mm longitudinal incision was made along both the left and right sides of the spine to squeeze out the kidneys and separate the renal pedicles to be clamped for 40 minutes. Reperfusion was done by removal of the clamps and kept for 24 hours. The virus was injected (10^11^ viral particles per injection) locally at the time of the end of the ischemia treatment.

### Measurement of renal function

At the end of the reperfusion (24 hours), blood samples were taken immediately by a heart puncture. The serum fraction was obtained after a 5 minutes’ centrifugation at 4,000 × g. The levels of serum creatinine and blood urea nitrogen were determined using corresponding diagnostic kits (ab65340, Abcam, Cambridge, MA, USA; EIABUN, BUN Colorimetric Detection Kit, ThermoFisher Scientific, Rockford, IL, USA).

### Isolation and differentiation of BMDMs

Bone marrow-derived macrophages (BMDMs) were isolated from 10-week-old male C57/Bl6 mice. The mouse bone marrow was withdrawn from the exposed marrow cavity with a 5ml syringe into an Iscove’s Modified Dulbecco’s Media (IMDM) containing 11% Fetal Bovine Serum (FBS), 8 units/ml penicillin and 80 mg/mL streptomycin. BMDMs were obtained after culturing in IMDM containing 11% FBS, 8 units/ml penicillin, 80 mg/mL streptomycin and 10 ng/ml macrophage colony stimulating factor (M-CSF) for 7 days at 37℃ and 5% CO_2_. M1 macrophages were induced by 100 ng/ml LPS and 20 ng/ml interferon gamma (IFN-ɣ) for 24 hours, while M2 macrophages were induced by 20 ng/ml IL-4 (19).

### Measurement of macrophage phagocytosis

Phagocytosis was measured by a specific assay (ab211156, Abcam). Differentiated M1 mouse macrophages were seeded at 20,000 cells/well overnight in a 96-well plate, after which 1,000,000 Zymosan particles were added for a 30 minutes’ phagocytosis process. Zymosan intake by macrophages was measured according to the protocol.

### Plasmids and adeno-associated virus

Mouse CD86 and CD163 promoters were cloned using mouse genomic DNA as templates, with information from UniProtKB/Swiss-prot. Complete coding sequence for Clec7a was obtained from the cDNA generated from mouse macrophages. Si-RNA for Clec7a was purchased from Santa Cruz (sc-63277). A scramble sequence (SCR) was used as a control for the transgenes. Lipofectamine 3000 (Invitrogen, CA, Carlsbad, USA) was used for generating AAVs (serotype 2, Vector Biolabs, Malvern, PA, USA).

### Flow cytometry

The mouse kidney tissue was digested with PBS with 0.2% trypsin (Invitrogen) and 5mg/ml DNase (Invitrogen) for 25 minutes to obtain a single cell fraction for fluorescence activated cell sorting (FACS). Macrophages were isolated based on double positive immunofluorescence for CD68 (with a FTIC-conjugated anti-CD68 antibody, #562117, Becton-Dickinson Biosciences, San Jose, CA, USA) and CD11b (with an Cy5-conjugated anti-CD11b antibody, #553311, Becton-Dickinson Biosciences). CD68+CD11b+ macrophages were further separated by their positivity for CD163 (with an Cy5-conjugated anti-CD163 antibody, #563887, Becton-Dickinson Biosciences) and Clec7a (with an BV421-conjugated anti-Clec7a antibody, #749795, Becton-Dickinson Biosciences). The flow cytometry data were analyzed and presented by Flowjo (Flowjo LLC, Ashland, OR, USA).

### EILISA

Protein values were determined by the corresponding ELISA kits for iNOS, TNFα, IL-1β, ARG1, TGFβ1, VEGF-A and IL-4 (R&D Biosystem, Beijing, China).

### Measurement of cell growth, invasion and migration

Cell growth was determined by a CCK-8 assay (Millipore, Bedford, MA, USA). Cell invasion and migration were done after 28 hours’ culture using xCELLigence RTCA DP Cell Invasion and Migration (CIM) Assay kits. For measurement of cell migration, CIM-Plate 16 was assembled, equilibrated in 37°C incubator for 1 hour. Afterwards, cells were added to the CIM-Plate 16 for 1 hour to allow attached. Afterwards, the CIM-Plate 16 was loaded into an xCELLigence RTCA DP instrument for 28 hours. Next, the data acquisition was stopped and the migrated cells on the underside of the membrane were stained and measured. For measurement of cell invasion, the upper chamber was coated with Matrigel and placed in a 37°C incubator for 6 hours. CIM-Plate 16 was then assembled, equilibrated in 37°C incubator for 1 hour. Afterwards, cells were added to the CIM-Plate 16 for 1 hour to allow attached. Afterwards, the CIM-Plate 16 was loaded into an xCELLigence RTCA DP instrument for 28 hours. Next, the data acquisition was stopped and the invaded cells were stained and measured.

### Statistical analysis

Statistical analysis was performed using one-way analysis of variance (ANOVA) test (GraphPad Software, version 9, Inc. La Jolla, CA, USA). “*” represents significance (p<0.05), while “ns” represents no significance (p>0.05). The group number (n) was determined by Power test, and n=4~6 was used. Individual values were shown in the figures.

## Results

### Bioinformatics show significant increases in Clec7a in renal macrophages after IRI-AKI

**Figure 1:**
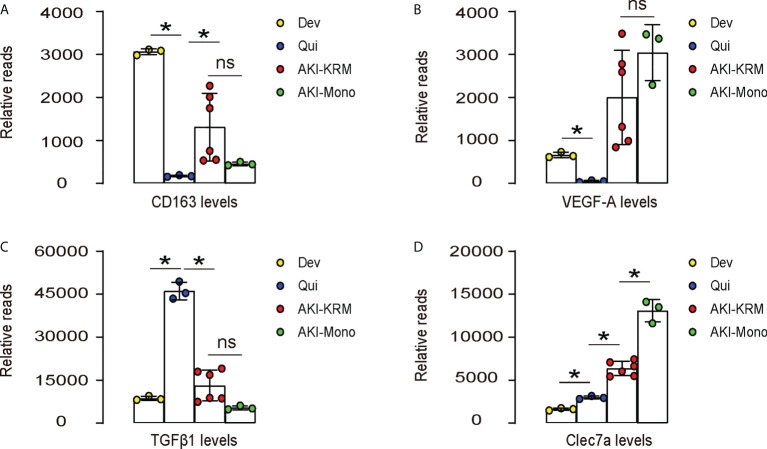
Bioinformatics show significant increases in Clec7a in renal macrophages after IRI-AKI. **(A–D)** Data were obtained from GEO database GSE121410, which recorded the analysis on mouse renal macrophages at development (Dev), quiescent status (Qui) and during AKI. AKI-macrophages were further separated as resident macrophages (AKI-KRM) and circulating monocytes -derived macrophages (AKI-mono). Transcript reads for CD163 **(A)**, VEGF-A **(B)**, TGFβ1 **(C)** and Clec7a **(D)** were shown. *p<0.05. ns, no significant.

The role of macrophages in AKI have been recently acknowledged. In a very nice study by Lever et al. (9), renal macrophages at development, quiescent status and during AKI were separated and analyzed in mice. We re-investigated the data that were published at the GEO database GSE121410. To our interest, we found that CD163, a M2 macrophage marker, was highly expressed in the developing kidney, but greatly leveled down in the quiescent adult kidney, suggesting that M2-polarized macrophages may play roles in the organogenesis of kidney (**Figure 1A**). The levels of CD163 in renal macrophages after AKI were, however, significantly increased in both resident macrophages (AKI-KRM) and circulating monocytes -derived macrophages (AKI-mono), suggesting that M2-polarized macrophages may play roles in the pathological progression of AKI (**Figure 1A**). Next, we analyzed the levels of vascular endothelial growth factor A (VEGF-A) in different macrophage populations. We found that VEGF-A was highly expressed in the developing kidney, but greatly leveled down in the quiescent adult kidney, suggesting that VEGF-A in macrophages may play roles in the vascular formation of the developing kidney (**Figure 1B**). The levels of VEGF-A in renal macrophages after AKI were, however, significantly increased in both AKI-KRM and AKI-mono, exceeding the levels of VEGF-A in the renal macrophages from the developing kidney, suggesting that macrophage-derived VEGF-A may play critical roles in the tissue repair and angiogenesis after AKI (**Figure 1B**). Interestingly, transforming growth factor β 1 (TGFβ1), a key pro-fibrotic factor, was even lower in AKI-KRM and AKI-mono, compared to the renal macrophages at quiescence, suggesting that macrophages may not the major source of the pro-fibrotic factors after AKI (**Figure 1C**). Lastly, we found that Clec7a, a factor associated with innate immune responses to pathogens and the phagocytotic properties, were significantly increased in the AKI-KRM and AKI-mono, compared to the renal macrophages at quiescence, suggesting its involvement in the regulation of post-AKI tissue response and repair by macrophages (**Figure 1D**).

### Differential expression of Clec7a in renal macrophages at quiescence and during AKI

**Figure 2:**
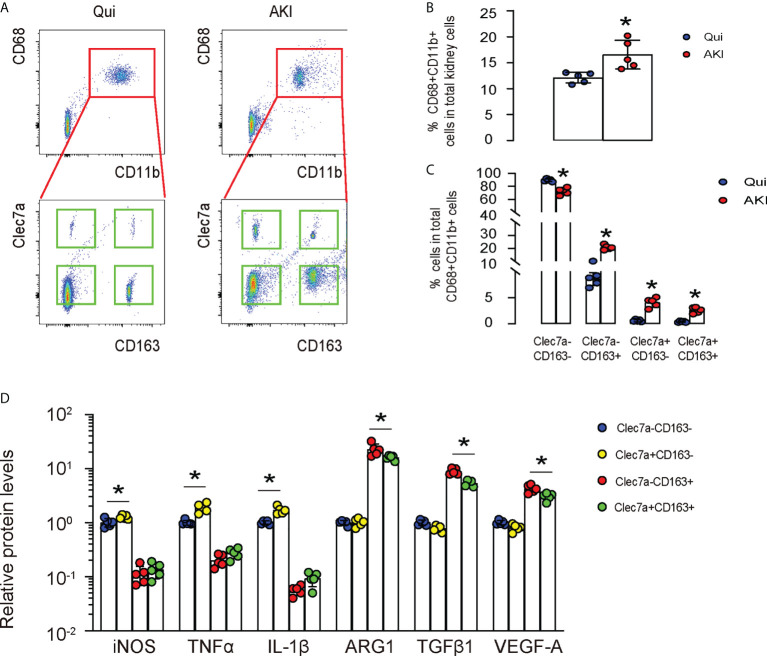
Differential expression of Clec7a in renal macrophages at quiescence and during AKI. **(A–C)** In a mouse renal ischemia/reperfusion injury (IRI) model for AKI, we isolated renal macrophages based on their double positivity for CD68 and CD11b, and then further separated them based on their positivity for CD163 and Clec7a to obtain 4 populations: Clec7a+CD163- M1 macrophages, Clec7a-CD163- M1 macrophages, Clec7a+CD163+ M2 macrophages and Clec7a-CD163+ M2 macrophages, shown by representative flow charts **(A)**, and by quantification for the percentage of CD68+CD11b+ macrophages **(B)** and of 4 different subpopulations **(C)**. **(D)** ELISA for some genes associated with macrophage polarization and function. *p<0.05.

Next, we used a renal ischemia/reperfusion injury (IRI), a mouse model for AKI, to study the importance of Clec7a in macrophages. First, we digested the kidney from mice with/without AKI, and sorted renal macrophages based on their double positivity for CD68 and CD11b (upper panels in **Figure 2A**). Next, the CD68+CD11b+ macrophages were further separated based on their positivity for CD163 and Clec7a (lower panels, **Figure 2A**). We found that renal macrophages could be further classified into Clec7a+CD163- M1 macrophages, Clec7a-CD163- M1 macrophages, Clec7a+CD163+ M2 macrophages and Clec7a-CD163+ M2 macrophages, representing distinct macrophage populations (**Figure 2A**). The percentage of CD68+CD11b+ macrophages in the kidney was slightly but significantly higher after AKI (**Figures 2A, B**). Moreover, the percentage of Clec7a-CD163- M1 macrophages in the kidney was significantly reduced after AKI (**Figures 2A, C**), while the percentage of Clec7a+CD163- M1 macrophages, Clec7a+CD163+ M2 macrophages and Clec7a-CD163+ M2 macrophages in the kidney were all significantly increased after AKI (**Figures 2A, C**). In order to understand the difference and functionality of these 4 renal macrophage subpopulations, we analyzed some key genes associated with macrophage polarization and function by ELISA. We detected significant higher levels of inducible nitric oxide synthase (iNOS), tumor necrosis factor alpha (TNFα) and IL-1β in the Clec7a+CD163- M1 macrophages compared to the Clec7a-CD163- M1 macrophages, suggesting that Clec7a may increase the M1-associated phenotype and cytokine production in M1 macrophages (**Figure 2C**). On the other hand, we detected significant lower levels of arginase 1 (ARG1), TGFβ1 and VEGF-A in the Clec7a+CD163+ M2 macrophages compared to the Clec7a-CD163+ M2 macrophages, suggesting that Clec7a may reduce the M2-associated phenotype and cytokine production in M2 macrophages (**Figure 2C**).

### Clec7a does not alter polarization of M1 or M2 macrophages

**Figure 3:**
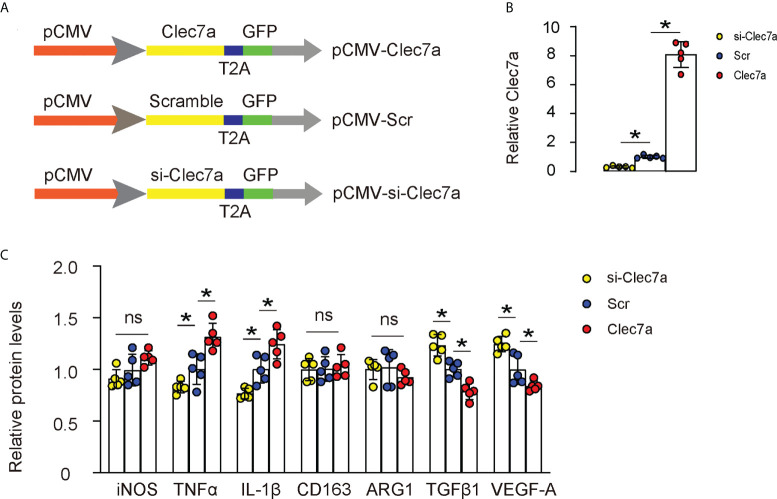
Clec7a does not alter polarization of M1 or M2 macrophages. **(A)** Schematic of generating plasmids carrying Clec7a or si-Clec7a or scramble control under a cytomegalovirus (CMV) promoter. **(B)** ELISA for Clec7a levels. **(C)** ELISA for iNOS, TNFα, IL-1β, CD163, ARG1, TGFβ1 and VEGF-A. *p<0.05. ns, no significant.

Next, we examined whether Clec7a may alter polarization status of M1 or M2 macrophages. We generated plasmids carrying Clec7a or si-Clec7a under a cytomegalovirus (CMV) promoter (**Figure 3A**). Transfection of mouse macrophages by Clec7a significantly increased Clec7a levels, while transfection of mouse macrophages by si-Clec7a significantly decreased Clec7a levels by ELISA (**Figure 3B**). Of note, Transfection of mouse macrophages by Clec7a significantly increased levels of TNFα and IL-1β, and significantly decreased levels of TGFβ1 and VEGF-A, while transfection of mouse macrophages by si-Clec7a significantly decreased levels of TNFα and IL-1β, and significantly increased levels of TGFβ1 and VEGF-A (**Figure 3C**). However, the levels of iNOS, CD163 and ARG1 were unchanged by Clec7a modifications (**Figure 3C**), suggesting that Clec7a does not alter polarization status of M1 or M2 macrophages.

### Clec7a increases phagocytic potential of M1 macrophages

**Figure 4:**
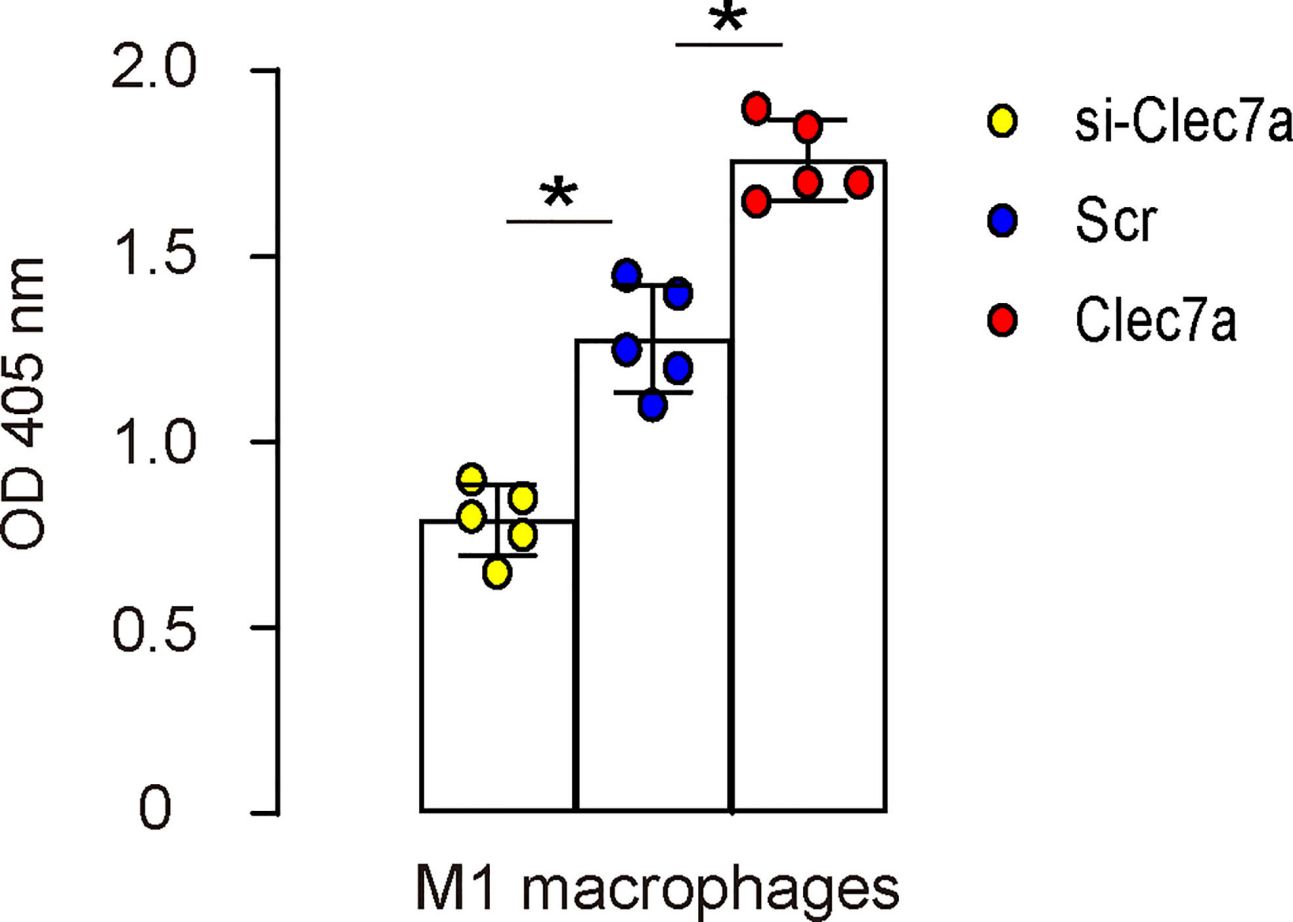
Clec7a increases phagocytic potential of M1 macrophages. A phagocytosis assay using Clec7a or si-Clec7a transfected LPS/IFN-ɣ-primed M1 macrophages. *p<0.05.

Since our data showed that Clec7a+CD163- M1 macrophages expressed higher iNOS, TNFα and IL-1β (**Figure 2D**), and since Clec7a increased TNFα and IL-1β in mouse macrophages (**Figure 3C**), we hypothesized that Clec7a may alter the phagocytosis of M1 macrophages. To prove it, we did a phagocytosis assay using Clec7a or si-Clec7a transfected LPS/IFN-ɣ-primed M1 macrophages. We found that transfection of mouse M1 macrophages by Clec7a significantly increased their phagocytosis, while transfection of mouse M1 macrophages by si-Clec7a significantly decreased their phagocytosis (**Figure 4**). Thus, Clec7a increases phagocytic potential of M1 macrophages.

### Clec7a depletion in M2 macrophages increases cell proliferation and migration

**Figure 5:**
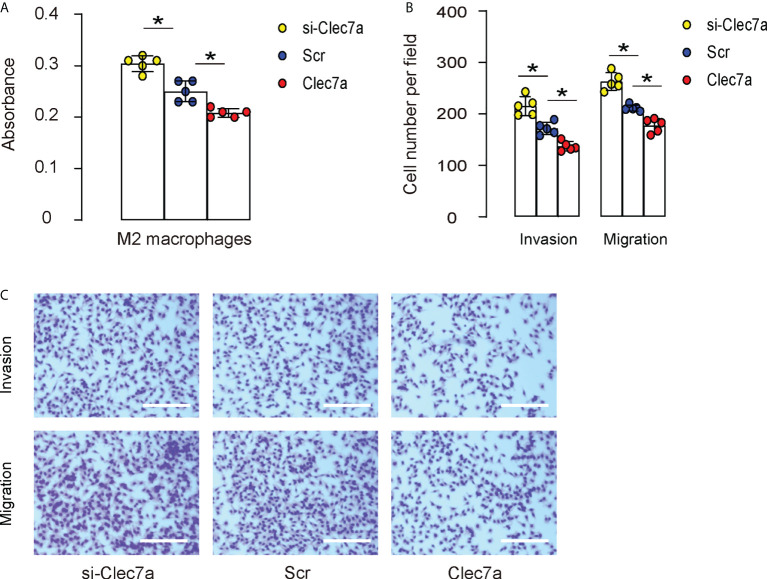
Clec7a depletion in M2 macrophages increases cell proliferation and migration. Cell growth, invasion and migration were measured in Clec7a or si-Clec7a transfected IL-4-primed M2 macrophages. **(A)** CCK-8 assay. **(B, C)** Cell invasion and migration assay, shown by quantification **(B)** and by representative images **(C)**. *p<0.05. Scale bars are 150µm.

Since our data showed that Clec7a-CD163+ M2 macrophages expressed higher ARG1, TGFβ1 and VEGF-A (**Figure 2D**), and since Clec7a decreased TGFβ1 and VEGF-A in mouse macrophages (**Figure 3C**), we hypothesized that Clec7a may reduce the repairing potential of M2 macrophages. To prove it, we measured the growth and invasion/migration potential of Clec7a or si-Clec7a transfected IL-4-primed M2 macrophages. We found that transfection of mouse M2 macrophages by Clec7a significantly decreased their growth (**Figure 5A**), invasion and migration (**Figures 5B, C**), while transfection of mouse M2 macrophages by si-Clec7a significantly increased their growth (**Figure 5A**), invasion and migration (**Figures 5B, C**). Thus, Clec7a depletion in M2 macrophages increases macrophage proliferation and migration.

### Combined expression of Clec7a in M1 macrophages and depletion of Clec7a in M2 macrophages significantly improve renal function after IRI-AKI

**Figure 6:**
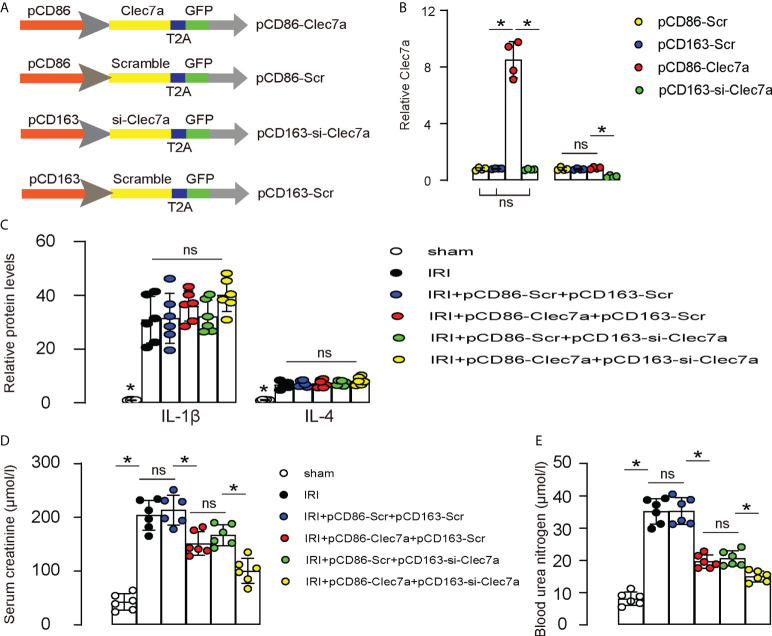
Combined expression of Clec7a in M1 macrophages and depletion of Clec7a in M2 macrophages significantly improve renal function after IRI-AKI. **(A)** Schematic to show generation of AAVs carrying Clec7a under a M1-specific CD86 promoter (pCD86-Clec7a) and AAVs carrying si-Clec7a under a M2-specific CD163 promoter (pCD163-si-Clec7a). Corresponding Scr controls were also generated. **(B)** ELISA for Clec7a levels in M1/M2 macrophages. **(C–E)** Mice were allotted into 6 groups. 1) Group 1: Sham, 2) Group 2: IRI, 3) Group 3: IRI and *in situ* administration of AAVs carrying pCD86-Scr and AAVs carrying pCD163-Scr, 4) Group 4: IRI and *in situ* administration of AAVs carrying pCD86-Clec7a and AAVs carrying pCD163-Scr, 5) Group 5: IRI and *in situ* administration of AAVs carrying pCD86-Scr and AAVs carrying pCD163-si-Clec7a, 6) Group 6: IRI and *in situ* administration of AAVs carrying pCD86-Clec7a and AAVs carrying pCD163-si-Clec7a. **(C)** ELISA for macrophage-derived cytokines, IL-1β and IL-4. **(D, E)** Renal function was measured 24 hours after IRI, by serum creatinine **(D)** and by blood urea nitrogen **(E)**. *p<0.05. ns, no significant.

Since our *in vitro* suggests that expression of Clec7a in M1 macrophages may increase their phagocytosis, while depletion of Clec7a in M2 macrophages may increase their repairing potential, we assessed the effects of these two approaches on the recovery of renal function after IRI-AKI in mice. To allow specific expression of Clec7a in M1 macrophages and specific expression of si-Clec7a in M2 macrophages, we generated AAVs carrying Clec7a under a M1-specific CD86 promoter (pCD86-Clec7a) and AAVs carrying si-Clec7a under a M2-specific CD163 promoter (pCD163-si-Clec7a). Corresponding Scr controls were also generated (**Figure 6A**). We found that pCD86-Clec7a significantly increased Clec7a levels in M1 macrophages, but not in M2 macrophages, while pCD1163-si-Clec7a significantly decreased Clec7a levels in M2 macrophages, but not in M1 macrophages, confirming the quality and specificity of these AAVs (**Figure 6B**). Next, mice were allotted into 6 groups. 1) Group 1: Sham, 2) Group 2: IRI, 3) Group 3: IRI and *in situ* administration of AAVs carrying pCD86-Scr and AAVs carrying pCD163-Scr, 4) Group 4: IRI and *in situ* administration of AAVs carrying pCD86-Clec7a and AAVs carrying pCD163-Scr, 5) Group 5: IRI and *in situ* administration of AAVs carrying pCD86-Scr and AAVs carrying pCD163-si-Clec7a, 6) Group 6: IRI and *in situ* administration of AAVs carrying pCD86-Clec7a and AAVs carrying pCD163-si-Clec7a. Renal levels of some cytokines associated with macrophages were determined, showing alterations by the corresponding treatments (**Figure 6C**). Renal function was measured 24 hours after IRI, showing significant improvement in serum creatinine (**Figure 6D**) and blood urea nitrogen (**Figure 6E**) by either expression of Clec7a in M1 macrophages or depletion of Clec7a in M2 macrophages, but the combined usage of two approaches generated significantly better outcome (**Figures 6D, E**). Together, Combined expression of Clec7a in M1 macrophages and depletion of Clec7a in M2 macrophages significantly improve renal function after IRI-AKI.

## Discussion

Repair after IRI mainly includes two processes: the solution of the local inflammation and the tissue regeneration mainly through TECs (20). Infiltrating inflammatory cells, in particular macrophages, play an important role in AKI initiation and repair-associated cell proliferation (21). Macrophages not only induces the apoptosis of TECs by secreting inflammatory cytokines and participates in the early injury during IRI, but also participates in the repair of renal tubules after IRI by regulating the inflammatory immune response (22). IRI can induce apoptosis or necrosis of TECs, resulting in decreased tubular integrity and interstitial inflammatory cell infiltration (23). At beginning, pro-inflammatory M1 macrophages was recruited to the kidney, and anti-inflammatory M2 macrophages predominated later on (24). Our model does not allow lineage tracing of resident macrophages versus circulation-derived monocytes/macrophages. Use of some markers to distinguish these two populations is not reliable, since macrophages alter their phenotypes in the progression of IRI, and during their infiltration to kidney from circulation. Thus, the contribution of Clec7a+ cells from different origins to the solution of IRI is not clear, which could be addressed in the future studies.

Clec7a is a specifically expressed in innate immune cells, is believed to be involved in the innate immune responses to pathogens and the phagocytotic properties (14–16). Therefore, it is expected to be involved in the early phase of AKI to help removal of pathogens and dead cells. In this stage, M1 macrophages play more important roles and compromised functionality of M1 macrophages could result in suboptimal clearance of the debris, which is harmful for the cell/tissue regeneration and functional recovery at later phase (25). Thus, it is not surprising that increased Clec7a levels in M1 macrophages improves the outcome of functional recovery of the kidney after AKI. In the animal model, we used CD86 as a specific promoter for M1 macrophage. However, CD86 is low expressed in some non-macrophages, such as dendritic cells and lymphocytes, which is a limitation of the current study.

On the other hand, our data also showed that expression of Clec7a in M2 macrophages may compromise their repairing potential, which is likely due to its suppression on angiogenic factor VEGF-A and tissue remodeling factor TGFβ1. Although Clec7a did not obviously alter the polarization status of a macrophage, its fine regulation of genes associated with the function of macrophages could have effects on the progression of the pathological events occurring during AKI (20). Indeed, a time-sensitive phenotypic alteration in renal macrophages may exert a critical influence on the outcome of AKI (26). Here, we provide a novel strategy to boost clinical therapy for AKI.

## Data availability statement

The original contributions presented in the study are included in the article/supplementary material. Further inquiries can be directed to the corresponding authors.

## Ethics statement

The animal study was reviewed and approved by Fudan University Animal Care and Use Committee.

## Author contributions

YW, XL, XX, JY, XHC, XSC, JZ, BS, and XD are responsible for data acquisition and analysis; YW, BS, and XD are responsible for study conception and design, data acquisition and analysis; YW wrote the manuscript and all authors have read the manuscript and agreed with the publication. BS and XD are responsible for funding and are the guarantee of the study.

## Funding

This study was supported by National Natural Science Foundation of China (NO: 81871598), Shanghai Municipal Key Clinical Specialty (NO: shslczdzk02501) and Shanghai “Science and Technology Innovation Plan” Yangtze River Delta Scientific and Technological Innovation Community Project (No. 21002411500).

## Conflict of interest

The authors declare that the research was conducted in the absence of any commercial or financial relationships that could be construed as a potential conflict of interest.

## Publisher’s note

All claims expressed in this article are solely those of the authors and do not necessarily represent those of their affiliated organizations, or those of the publisher, the editors and the reviewers. Any product that may be evaluated in this article, or claim that may be made by its manufacturer, is not guaranteed or endorsed by the publisher.
